# Implementing the UN Sustainable Development Goals: How Is Health Framed in the Norwegian and Swedish Voluntary National Review Reports?

**DOI:** 10.34172/ijhpm.2020.221

**Published:** 2020-11-23

**Authors:** Ida Lillehagen, Kristin M. Heggen, Göran Tomson, Eivind Engebretsen

**Affiliations:** ^1^Centre for Sustainable Health Care Education, University of Oslo, Oslo, Norway; ^2^Swedish Institute for Global Health Transformation (SIGHT), Royal Swedish Academy of Sciences and Presidents Office, Stockholm, Sweden.; ^3^Karolinska Institutet Stockholm, Stockholm, Sweden.

**Keywords:** Sustainable Development Goals, Voluntary National Reviews, Discourse Analysis, Framing Analysis, Nordic Region

## Abstract

**Background:** The United Nations (UN) Sustainable Development Goals (SDGs) are parts of an ambitious framework for global development, the 2030 Agenda. Voluntary national reviews (VNRs) are described as "cornerstones" in the followup system, which is premised on international sharing of knowledge and experience. Norway and Sweden are among the world’s most sustainable countries, aiming to be leaders in the implementation of the SDGs. The objective of this article is to investigate and compare how health is framed in the VNRs of these two high-income countries, and to discuss the implications of these framings for potential actions.

**Methods:** Discourse analysis inspired by the concept of ‘framing,’ which refers to the discursive presentation of an issue where certain problem definitions and solutions are privileged over others. Frames are structures that organise and direct attention to particular aspects of reality, and define what is seen.

**Results: **Our analysis demonstrates that in the Norwegian VNR (NVNR), the issue of health is simplistically framed, focusing on the favourable situation of the majority, thus providing weak grounds for transformative action. In the Swedish VNR (SVNR), health is framed to highlight health as inextricably tied to societal inequalities. This underscores the need for integrated political action and leadership to counteract structural differences with negative consequences for health.

**Conclusion:** Analysis of the two VNRs studied found a difference in how health is framed in these documents and these frames point to differences in approach and capacity to address health inequities and realise the holistic and integrative concept of health promoted in the 2030 Agenda. To realize the Agenda’s vision of "leaving no one behind" discourses of implementation that support the Agenda’s inclusive and holistic ambition must be developed. Further development of the follow-up and review system should acknowledge and address how frames can limit or enable integrative actions and are therefore important drivers of change.

## Background

**Key Messages**

** Implications for policy makers**

- The 2030 Agenda underscores integrated and cross-sectoral efforts to achieve the Sustainable Development Goals (SDGs). This article identifies tensions between the inter-sectoral nature of the SDGs and their reductionist metrics-based follow-up and review system, which provide weak grounds for transformative action for health equity.
- Policy-makers can utilize the presented results to ensure that future framings of health adequately reflects the 2030 Agenda’s transformative ambition and focus on leaving no one behind.
- Further development of the follow-up and review system should acknowledge and address how frames can limit or enable integrative actions.

** Implications for the public**

The 2030 Agenda emphasises the all-encompassing responsibility of realizing the Sustainable Development Goals (SDGs), calling upon actors from individual to global levels to “be the change.” Discourses that gloss over conflicting concerns between groups and issues provide weak ground for political engagement. There is a need for civil society groups to contribute to the debate on implementing the SDGs, and on scrutinising indicators to assess progress toward them. This article can contribute with insights relevant for critical public engagement and contestation.

In 2015, the United Nations (UN) General Assembly committed to 17 Sustainable Development Goals (SDGs) as part of an ambitious universal framework for global development: the 2030 agenda.^1^ The 17 SDGs are considered integrated and indivisible, forming a universal policy agenda that presents a plan of action for people, planet, and prosperity.

 Health is addressed in Goal 3: “Ensure healthy lives and well-being for all at all ages,” a formulation that reflects a broad definition of health as physical, mental and social well-being.^2^The 2030 Agenda stresses that the SDGs are “integrated and indivisible,” thus highlighting the interdependency of health and wider social determinants, as well as a cross-cutting focus on “leaving no one behind.” Scholars have argued that the 2030 Agenda’s holistic and integrated conception of health calls for an inter-sectorial “health in all-approach” to policy development where health is both a prerequisite for and outcome of sustainable development.^3-7^

 A central feature of the 2030 Agenda is the emphasis on implementation. SDG 17 aims to “strengthen the means of implementation and revitalize the global partnership for sustainable development.” The Agenda outlines a follow-up and review system to promote implementation and monitor progress. A key instrument of the system is the indicator framework developed by the Inter-Agency and Expert Group on SDG Indicators (IAEG-SDG), consisting of about 230 statistical indicators.^8^ Another central feature of the follow-up and review system is the ‘voluntary national review reports’ (VNRs). Reporting on the status of SDG implementation “based on statistical data, using SDG indicators to the extent possible and outlining the factors of success or failure to achieve progress,” the VNRs are described as: “the cornerstone of the follow-up and review system.”^9^ The purpose of the VNRs is to accelerate the implementation of the 2030 Agenda, as well as to: “strengthen policies and institutions of governments and to mobilize multi-stakeholder support and partnerships.”^10^VNRs are described as: “voluntary and country-led, will take into account different national realities, capacities and levels of development and will respect policy space and priorities.”^9^The VNRs will also serve as the basis for the regular reviews by the high-level political forum (HLPF). Per December 2019, 158 nations had presented a VNR.^10^

 As has been previously pointed out, implementation of the SDGs mainly relies on national responsibilities.^11,12^VNRs are developed by nations, and intended to outline the nation’s responses and progress towards goals’ achievement, as well as providing the basis for UN recommendations and “shared experiences.” Principles of national sovereignty and voluntariness in the follow up and review system provides nations with space to adapt their follow up to national capacities and circumstances, but obviously also to selectively focus on issues that are aligned with national interest, while avoiding challenging or contested issues.^12^ Lacking binding structures, the follow up and review system relies heavily on the essentially voluntary efforts of national governments (which may or may not be fully supportive of the SDGs) to develop VNRs that are fit for purpose. To integrate actions on the SDGs, VNRs need to use discourses with frames that facilitate the 2030 Agenda’s integrated and holistic ambition. Text production in the follow up system, and particularly the VNRs, are thus crucial tools in ensuring the realisation of the 2030 Agenda.

 Unlike the Millennium Development Goals, which specifically targeted less developed countries, the SDGs address the joint responsibility of *all* countries to work for a more sustainable future. The Nordic countries rapidly responded to this, aiming to be global leaders in the implementation of the 2030 Agenda.^13,14^ Sweden is ranked the most sustainable country in the world on the Sustainable Development Solutions Network SDG Index, whereas Norway is ranked number three.^15^ In March 2016, the Swedish government confirmed that its primary ambition is “for Sweden to be a leader in the implementation of the 2030 Agenda.”^16^ In January 2016, The Norwegian Prime Minister, Ms Solberg was appointed co-chair for the UN Sustainable Development Goals Advocacy Group, and Norway was one of the first countries to present a VNR. Considering these countries’ commitment to Agenda 2030, their VNRs may be seen as potentially norm-defining to the SDG follow up, as well as in global health more broadly.

 In this article we present an analysis of the Norwegian VNR (NVNR) from 2016,^17^ and the Swedish VNR (SVNR) from 2017,^18^ focusing on and comparing how health is framed in descriptions of and indicators chosen for the domestic implementation of the 2030 Agenda.

 Considering the key role of the VNRs in the follow-up and review system, scholars have so far granted these documents relatively little external and critical attention. We have identified some evaluations and analyses by external experts, mostly published by non-government organizations.^19-22^ In a review of the literature on VNRs (22 publications) published by the Overseas Development Institute, the authors find that many reports focus on whether or not specific themes are addressed in the VNRs.^22^ Less attention is given to *how* the issues are being addressed. For instance, various forms of content analysis are utilized to describe the prevalence of specific issues or principles, as well as connection between issues.^23,24^ In a background paper for the UN Committee for Development, Fukuda-Parr et al^25^ present a review of 43 VNRs, providing a good overview of important topics. However, they conclude, there is a need to complement such overarching evaluations with in-depth analyses of *how *nations present policy for implementation of the SDGs.

 The importance of understanding how policy is discursively presented, and how this, in turn, implies certain solutions over others, have been described in a stream of literature concerned with the framing of policy issues in global health.^26-32^ A perspective of policy-making as a constant discursive struggle over ideas and values allows us to analyse not merely how policy-makers solve problems, but also how they formulate and prioritize those problems in the first place – and conversely, how certain issues come to be defined as ‘non-problems,’ or simply not seen at all.^32^ For instance, in an analysis of the framing of inequality in the SDGs, Fukuda-Parr describes the politicization of the seemingly technical and objective process of choosing targets and indicators to measure inequality (SDG10).^33^ She illustrates how the choice of indicator was connected to attempts of framing the inequality agenda as inclusion, focusing on the poor and excluded, rather than on extreme inequality. She argues that the insertion of the shared prosperity measure in setting the target on vertical economic inequality (rather than distribution measures such as Gini or Palma ratio) was strategic to keep extreme poverty out of the SDGs. The analysis is also an illustration of how the SDGs can contain multiple framings that can align, overlap, differ and be in tension with each other.

 The study of framing of policy issues has increasingly gained relevance as the use of evaluations, such as rankings and performance reviews have proliferated in general,^34^ and specifically in global governance through goal setting.^35^Evaluations have become key documents in an “evaluation society” where reporting practices take new precedence in policy-making practices.^36^ As such, evaluation documents has become increasingly important documents for structuring what we talk about, what we think are important problems, and what we think we will do about these problems.^36,37^

## Methods

###  Design

 This article presents a comparative analysis of the discourses used in the NVNR and SVNR. Our analytical approach is inspired by the concept of ‘framing,’ which has been extensively used in discourse analyses of public policy development.^29,31,38,39^ It is defined by Entman^40^ as: “to select some aspects of a perceived reality and make them more salient in a communicating text, in such a way as to promote a particular problem definition, causal interpretation, moral evaluation, and/or treatment recommendation for the item described.” The discourse analysis in this article investigates how words and phrases can act as frames to create dominant understandings of problems and solutions, or more simply, how language can structure political thought and action. This article analyses discourses used in the NVNRs and SVNRs and how they authoritatively define and naturalise certain meanings of health, and how such meanings constrain or enlarge possibilities for action. Official discourses, ie, texts and utterings by agents that society generally imbues with political power and significance, formally instruct public policy and services. However, discourses are not seen as fully determining action in a causal relationship, but as a legitimising and reinforcing factor to political power.^41^As well, there may be tensions between the discourses and the political priorities governments wish to emphasise at international and national levels.^42^

###  Data Material 

 The NVNR, called “Initial steps towards the implementation of the 2030 Agenda,”^17^ was published in 2016, the first year after the launch of the Agenda. The document includes a summary (4 pages), an introductory part (3 pages), a presentation of all 17 goals in the Norwegian setting (21 pages), and a conclusion (2 pages).

 The SVNR, called “Sweden and the 2030 Agenda - Report to the UN High-Level Political Forum 2017 on Sustainable Development,”^18^ was published in 2017. The document includes a summary (4 pages), an introductory part (9 pages), a presentation of the goals (24 pages), a part presenting current work in Sweden to implement the Agenda (38 pages), and a part on continued work (3 pages).

 The analysis also led us to government documents (strategies and action plans) describing Norwegian and Swedish policy with implications for health. We also read speeches and newspaper articles where government representatives comment on work towards the SDG. The study is thus informed by a comprehensive background material.

###  Text Analysis

 The results were generated through four separate, but interrelated steps of analysis. IL, EE and KH, all of which have competency in discourse analysis, conducted the first two steps. The first step involved repeated and interchangeable reading of both documents in their totality to get a general sense of how the documents and marking excerpts where health was addressed directly and indirectly. In the second step, we searched the documents for keywords and phrases, such as health, well-being, welfare, leaving/left behind, inequality, equity, rights, marginal, and vulnerable. We made comprehensive tables with excerpts of every mentioning of health/healthy/unhealthy, to identify in which textual context and how health was described throughout the documents. We used colour-codes to visualise preliminarily identified dimensions in the descriptions of health in the national setting, such as rights, inequality, universality, intersectoral collaboration, references to national policy and action plans, universal/local, marginal/vulnerable groups, and individual/government responsibility. Comparison was an important asset in this phase, as the reading of one text informed the reading of the other in terms of generating awareness of topics, similarities and differences. The third step involved close reading of marked excerpts to analyse the discursive construction of issues, ie, how the text produces particular representations, giving the impression of “truth” through language and argument.^32,43^ We specifically looked for inclusion and exclusion of topics and creation of causal relations through the use of for instance wording, metaphors, frequency and sequence.^41^ We also analysed the degree of certainty in claims (facticity), looking at ways that the text authorises and generalise claims (universality) or moderate them.^44^ IL, EE, and KH collaboratively validated the first categorization of data. In the third step, we chose to apply the concept of framing to further refine and describe the differing representations of health. The fourth step of analysis involved writing and rewriting results to clarify and revise analytical points. GT provided crucial input to analysis, background and context in the third and fourth steps. All authors were involved in critically revising and improving the manuscript.

## Results

###  National Baselines: How Is the Issue of Health and Health-Related Challenges Introduced in the Norwegian and Swedish VNRs?

 Both the NVNRs and SVNRs address health in introductory sections where national benefits and challenges for implementation of the SDGs are presented. For instance, in the NVNR it is stated in a section called “Challenges at the national level” that: “Among the targets that are likely to remain the focus of political attention and policy development are those relating to sustainable consumption and production, health and education, equality, employment, and migration.”^17^ The paragraph further contains a list of challenges identified at the national level. One of the bullet points: “reducing non-communicable diseases and promoting mental health,” is part of SDG3. Several of the other prioritized bullet points can also potentially have positive impacts on health (eg, “eliminating all forms of violence against women and girls;” “reducing the proportion of young people not in employment, education or training; “sustaining income growth of the bottom 40% of the population at a rate higher than the national average;” “improving urban air quality”).^17^

 In the SVNR section: “Favourable starting position – and significant challenges,” the issue of health is introduced with the following statement: “Since 2003, there has been a national public health policy adopted by the Riksdag covering eleven target areas. The overarching goal adopted for this policy is to create societal conditions for good health on equal terms for the entire population. The Health and Medical Services Act (2017:30) stipulates that care shall be delivered with respect to the equal value of all humans and the dignity of every individual. Since Autumn 2016, gender-equal health has become one of the targets of the overall gender equality policy in Sweden.”^18^

 There are important differences in the documents’ initial framing of health. In the NVNR, health is addressed as one of several issues in a list. Health is placed together with “education” to form a pair: “health and education,” together with other issues that could be understood as policy areas, but also as societal domains, or as societal values. Furthermore, it is the *targets related to *[the issues] that *are likely* to remain focus of political attention and policy development. No information is provided about which targets that are seen as related to these issues, or the relationships between them, something that makes the introduction highly ambiguous as to how these issues are understood or will be further pursued. The ambiguity is further underscored by the use of language that does not provide a clear commitment to the issues (the word “likely”), and that seems conservative (the word “remain”) more than transformational. The following list of challenges that have been identified at the national level specifies non-communicable diseases and mental health. While both of these categories include conditions known as correlating to dimensions addressed in the other bullet points (eg, gender, income, education, geography), the document refrains to mention any connections between health challenges and other societal challenges.

 In the SVNR, health is introduced with a reference to existing public health policy as a key measure to “create societal conditions for good health on equal terms for the entire population.” The presentation highlights health as a fundamental human right, by reference to the “Health and Medical Services Act,” and by use of formulations such as “the equal value of all humans and the dignity of every individual.” Thus, the presentation clearly establishes a frame of health as a fundamental human right that is premised upon creation of societal conditions through public health policy.

 By comparison, we see that whereas the NVNR introduces several topics central in the 2030 Agenda (eg, health, education, equality) in the initial description of national challenges, the presentation does not describe how or why these issues pose a challenge, whether or how they are connected, or how they will be addressed. The result is an unclear and non-committal frame of national challenges, including the framing of health. In the SVNR, the section on national challenges establishes a frame of health that emphasises the fundamentally intrinsic nature of health and societal conditions, with a clear commitment to improving health through improving social, environmental and economic conditions, and to ensure health as a human right.

###  Leaving No One Behind?

 Under SDG3 in the NVNR, health in the Norwegian setting is described as follows: “The general health of Norwegians is good. Life expectancy, both for men and women, is comparatively high, and Norway has well-established public health policies and health services. The Sami people face some challenges relating to access to culturally adapted health and care services.”^17^

 Life expectancy in any given year shows how long we expect that a child born that year will live.^45^ The measurement reflects mortality in a population and is, therefore, an important indicator of public health.^45^ In the NVNR, life expectancy is used to underpin the claim that “the general health of Norwegians is good.” As an average measurement, life expectancy numbers privilege the many; they attempt to speak for all based on what is most frequent. In Norway, life expectancy numbers vary with socio-economic and demographic variables like place of living, level of education, and occupation, and the inequalities are increasing.45 Stating that the general health of Norwegians is good with a reference to an average measurement, and without commenting on the increasing inequalities hidden in these numbers, the NVNR constructs a category of “Norwegians” as a homogenous population with “good general health.” The average measurement thus has a double effect; it unifies the category of “Norwegians” as all-inclusive, and at the same time increases the universality and facticity of the claim that their health is good.^44,46^ The effect is that attention is drawn towards the good health of the many implying little need for transformational change.

 Minority groups and groups of particular vulnerability in the context of health are thus pushed to the margins in descriptions of health in the national setting – a marginalisation that seems to be systematic in the NVNR. We find that the situation of groups that facing particular challenges within the national context is systematically toned down. For instance, under SDG1 “End poverty in all its forms everywhere” it is stated: “Norway is already considered to fulfil target 1.3 (implement nationally appropriate social protection systems and measures for all, including floors, and by 2030 achieve substantial coverage of the poor and the vulnerable). The same is the case for the other SDG 1 targets. Free or low-cost access to health, education and welfare services make the situation for low-income groups in Norway better than that in many other countries. The Norwegian Government will work to make Norway a country with a low level of income disparity and minimal poverty.”^17^

 Similarly, under SDG10 “Reduce inequality within and among countries” it is stated: “Income inequality in Norway is lower than in almost all other countries. High employment and relatively low unemployment mean that a large percentage of the population participates in income-generating work. Cash transfers provide compensation for loss of income due to illness, disability, old age, unemployment, etc. In addition, the national and local authorities provide free access for all to education, health, nursing and care.”^17^

 Interestingly, the text seems to refrain from mentioning people living under difficult conditions in Norway at all. “Low-income groups in Norway” is mentioned, but without any specification, and in a sentence describing their situation as “better than in many other countries.” By stating that: “a large percentage of the population participates in income-generating work,” the text again draws attention to the majority. The next sentence on cash transfer as compensation for loss of income due to illness, disability old age, unemployment etc renders the affected minority groups literally invisible by grammatically leaving out the recipients. By comparing low-income groups to other countries instead of to the majority in Norway, or by simply leaving them unmentioned, the NVNR systematically draw attention away from groups that suffer from disadvantageous conditions in Norway.

 Turning to the SVNR, we find that it too introduces life expectancy numbers in the first paragraph under goal 3: “In 2015, the average life expectancy for women was 84 years and for men 80.4. […] The remaining number of years at the age of 30 is increasing for the population as a whole but is several years greater in the group with post-secondary education than in the group with pre-secondary education. This is true of both women and men. These differences have increased over the past ten years.”^18^

 In this quote, the inequality dimension is immediately introduced as a function of pointing to the *insufficiency* of the average numbers to describe the population as a whole. Gender and level of education are introduced as to lines along witch health inequality is distributed. For instance, the formulation “for the population as a whole” is used to describe a tendency of increasing numbers of remaining years at 30 but is modified with inequalities by level of education in a way that highlights the determinants of inequality rather than the general tendency of the many.

 In subsequent descriptions, the SVNR identifies some groups facing particular vulnerability related to the health targets and the health system in the Swedish context. Yet, the clearest focus on inequality in health is communicated in the concluding section which states that: “The challenges for Sweden lie in taking measures for health equity, including the reduction of disparities in health and well-being between different groups in society and improving quick and equal access to healthcare for all who are in need of it […] There are also particular challenges regarding differences in both mental and physical health between different groups of the population, mainly between people with different levels of education and depending on gender. Furthermore, there are differences regarding these factors between LGBT [lesbian, gay, bisexual, and transgender] persons, people with disabilities, foreign-born persons, national minorities and indigenous peoples, and the population as a whole.”^18^

 A key difference in the framing of health in the two VNRs is thus the focus, or lack of such, on inequality and groups of vulnerability in the context of health. Foregrounding the majority, the NVNR can highlight the comparatively beneficial situation in Norway. Those furthest behind in Norway, ie, groups that face systematic disadvantage across a range of dimensions are almost not present in the document at all. Marginalisation is mentioned in very general terms and disconnected from any policy areas (eg, “all people must have the same opportunities for personal development, participation and self-realisation, irrespective of their gender, ethnicity, race, religion or belief, indigenous identity, sexual orientation or disability”^17^), and concerning international settings. This framing is in stark contrast to the SVNR, in which inequality in health is systematically highlighted as a structural problem.

###  Healthy Lives and Well-being? Mechanisms of Simplification and Selectivity

 The NVNR identifies non-communicable diseases and mental health as national challenges,^17^ Under SDG2 “End hunger, achieve food security and improved nutrition and promote sustainable agriculture” it is stated: “Nutritional disorders – primarily related to an unhealthy diet and lack of physical activity – are a challenge. In 2017, the Norwegian Government will submit a comprehensive action plan on healthy diets. The plan will be based on collaboration between the ministries responsible for children and equality, fisheries, agriculture, education, integration, and climate change and environment.” In comparison, the SVNR describes the same problem as follows: “Data instead points to an increase in overweight and obesity in society over the past decade. This increase is tangible in the age group 16-29 years. More than half of all adults in Sweden are overweight or obese. However, there are elderly people who suffer from malnutrition. There are differences and inequalities in eating habits and health that are closely associated with socio-economic situation, educational level and income.”

 A key difference in the framing of nutritional issues is that the NVNR presents the problem as general – as neutral along lines of inequality. Combined with the emphasis on unhealthy diets and lack of physical activity, the problem is framed as primarily an individual matter and responsibility. The close association to socio-economic situation, level of education and income that is made explicit in the SVNR is omitted.

 The example is of importance for the document’s framing of health for two reasons. First, while it is a concrete example of the integrated relationship between goals, it also constitutes a simplification, by framing the complex causes of nutritional disorders as a predominantly individual problem. Second, it is stated that nutritional disorders will be addressed by a comprehensive and intersectoral action plan, yet the individual problem definition undermines this and provides a highly ambiguous mandate for such collaborations. As one of the very few concrete examples of the prioritised non-communicable diseases, the description of nutritional disorders is telling for the framing of health in the NVNR, failing to underscore important health inequalities and complex association between societal and individual determinants that could give concrete direction for concerted efforts to address them.

 The simplification of health is furthered under SDG3 where it is stated that 4 of 13 targets, 3.3, 3.4, 3.5, and 3.9 (see Supplementary file 1) are of particular concern in a national setting based on national mortality and morbidity data.^17^ Using data that relies on biomedical categories and a health system authorised to count is in itself a narrow basis and foregrounds a disease-oriented conception of health. The word “prioritise,” meaning to grade importance or arrange an order of action has in many instances positive connotations as a symbol of a proactive and efficient attitude. However, the nine targets that are not prioritised are de-selected, ie, left out of subsequent descriptions. Prioritising, which in many instances can be necessary and highly legitimate is in this case used as a mechanism to select a narrow set of indicators and reinforce a disease-oriented conception of health in the NVNR.

 The SVNR’s description of health in Sweden follows the structure of SDG3’s targets and indicators. Most of the thirteen targets under goal 3 (except 3.9., 3C and 3D) are commented with numbers and additional information. In these comments, challenging issues related to the targets and indicators are addressed. For instance, under target 3.1. (maternal mortality ratio), where Sweden meets the suggested indicators, it is stated: “There are structural differences in care and treatment between women and men in the healthcare system. The Government is, therefore, making several major investments to strengthen women’s health and develop the care that is specifically aimed at women, including midwifery.”^18^

 Several comments in further descriptions explicate relevant challenging issues, particularly enhancing groups of vulnerability in the context of given targets, subjective health-related experiences in connection to membership in such groups, as well as structural discrimination. For instance, as a comment to target 3.8 (universal health coverage), it is stated that “the challenges for Sweden lie in taking measures for health equity, including the reduction of disparities in health and well-being between different groups of society and improving quick and equal access to healthcare for all who need it.”^18^ Importantly, the SVNR’s recurrent and explicit focus on groups suffering from inequalities in health and health services, highlight the inextricable relationship between health and societal determinants.

 That is, in the NVNR several mechanisms of exclusion, simplification and selectivity contribute to a biomedical framing of health that directs attention away from inequalities in the Norwegian population, and reduces opportunities to realise the Agenda’s integrated and holistic ambitions. Focusing on the majority enables a favourable picture implying that little action is necessary, something that is enhanced by a conception of health that privileges biomedical categories of health and is presented in isolation from societal structures and inequality. Ultimately, the framing of health in the NVNR provides a weak foundation for integrated and transformational implementation of health-related goals in Norway.

 The SVNR in contrast clearly establishes a frame of health in which health is seen as both a determinant and outcome of sustainable development, intrinsically connected to wider societal structures and maintaining the focus on “those furthest behind.” Health is more broadly conceptualised as an integral part of life as lived within societal structures, and not an issue of presence or absence of disease, thus establishing an implementation discourse in which inter-sectoral collaboration can be pursued.

## Discussion and Conclusions

 A close analysis of the framing of health in descriptions of national implementation of the SDGs in the NVNR and SVNR demonstrates how, by use of language and rhetoric radically different frames of health are established in the NVNR and SVNR respectively.

 The NVNR represents an interesting misalignment to the framing of health that has been prevailing and politically uncontroversial in Norwegian health policy for many years. Norway has long traditions of promoting views of health as inseparably tied to overall societal structures, something that is also reflected in white papers outlining national health policy and strategies going back many years. In 2019 the public health report: “Good lives in a safe society” defined reduction of health inequality as one of three main goals.^47^ Norway has also been an influential actor in the establishment and funding of international health institutions to advocate this view globally.^48^

 Why does the NVNR seem to be misaligned with a more societal framing of health that has enjoyed broad political support in Norwegian health policy for many years? Agenda 2030 signals a shift to a universal understanding of joint global challenges, thus requiring that high-income countries understand not only their own strengths but also their own shortcomings in relation to the goals. National ownership of the SDGs has been deemed a key challenge for the Nordic countries where many of the SDG indicators are already achieved.^13^ In light of this, the observed shift of frame in the NVNR could be understood as a reflection of being early days in the process of embedding the SDGs nationally, given that Norway presented its VNR just one year after the adoption of the Agenda.

 However, other aspects suggest this can only partially explain the framing of health in the NVNR. For instance, whereas the NVNR repeatedly states Norwegian commitment to the 2030 Agenda’s cross-cutting focus on inequalities and vulnerable groups, our results demonstrate that apparent indicator-fulfilment can be used to obscure remaining national challenges. The majority-focused framing may also be an expression of political unwillingness to focus on groups that, in global comparison, are in a better situation. This perspective was part of the negotiations of the 2030 Agenda. In fact SDG10: “Reduce inequalities within and among countries” was, according to Fukuda Parr^49^ resisted particularly by high-income countries because: “Goal 10 requires a reversal rather than acceleration of current trends in many countries, and it is relevant to all countries, regardless of the level of income. As such, it draws international attention to the need for wealthy, ostensibly ‘developed’ countries to address issues which draw the model they have followed into question.”^49^ In light of this, the framing of health in the NVNR could also be interpreted as an expression of political resistance to fundamental revisions of the existing system. The following quote from the NVNR is a signal in that direction: “Because most of the obvious instruments for reducing inequality have already been adopted, it may be challenging for Norway to reduce inequality further.”^17^ Moreover, in 2019, 30 civil society organisation leaders in Norway signed a debate article arguing that Norway’s commitment to the SDGs seems to be greater on the international scene and that domestic efforts need to be clarified and improved.^50^ On this backdrop, the identified framing of health in the NVNR could also be seen as reflecting a Norwegian understanding of the Agenda as internationally transformational, whereas national implementation will take place within the limits of existing systems and models.

 VNRs should, as noted by Bexell and Jönsson^42^ in a recent article on the politics of global evaluation reports, also be understood in light of sitting governments’ political ideologies. In Norway, Prime Minister Solberg (Conservative Party) has led a blue coalition of centre-conservative parties since 2013, which is currently consisting of the Conservative Party, the Christian-Democratic Party, and the Liberal Party. The Progress Party resigned from government in January 2020. The Conservative Party, which is the biggest party in government, has traditionally been committed to free-market policies, tax cuts and relatively little government involvement in the economy. The government is supporting the continued existence of the Norwegian welfare state, but has made the concept of a ‘sustainable welfare state’ a central term in their political platform.^51^ Civil society organisations have argued that the term has been used to justify reduced public spending and cuts in the welfare system. A report by the Norwegian Confederation of Trade Unions^52^ provides an overview of suggested and implemented welfare cuts, for instance in the disability pension, the work assessment allowance, the unemployment benefit, and the transitional benefit, which they conclude, contribute to increasing inequality. The report from the Ministry of Health and Care Services on the work towards the SDGs, in the proposed national budget for 2021^53^provides a general overview over some of the measures taken to achieve the prioritised targets under SDG3. For instance, the follow up and renewal of the national strategy for non-communicative diseases includes new action plans on physical activity and suicide prevention. How and to what extent these policies can contribute to national realisation of the 2030 Agenda should be subjected to further analysis.

 In Sweden, Stefan Löfven (Social Democratic Labour Party) has been Prime Minister since 2014. When the SVNR was published in 2017, he led a minority coalition government with the Green Party. Since January 2019 Löfven still leads a government consisting of the Social Democratic Party and the Green Party,^54^ but with only 33% of the Swedish Parliament seats, the government is regarded one of the weakest in Swedish history, relying on support from The Centre Party and The Liberals. Social democratic ideology and ideals of equality and redistribution is thus under strong pressure from the opposition of the Moderate Party, the Christian Democrats, the Sweden Democrats and the Left Party.

 According to Bexell and Jönsson’s^42^ study of the process of preparing the SVNR, the process was characterised by a political wish to live up to Sweden’s status as an “SDG champion.” Notably, they found that the HLPF report involved a “strong symbolic dimension supporting an identity formation,” especially with regards to the all-inclusive and cross-sectoral approach. The selection of participants to the official Swedish HLPF delegation is highlighted as having a strong symbolic dimension as supporting identity formation, emphasising cooperation with all sectors of society. The delegation consisted in representatives from government agencies, municipalities, trade unions, civil society organisations, parliamentarians, private business and academia. However, as noted by some informants, the cross-sectoral collaboration was not very well-anchored with the involved partners. Although the informants were generally positive, delegation members were not clear on what they were supposed to do during the HLPF, where the VNRs are presented, and did not fully understand expectations on their participation.

 An important aspect of the different frames in the two VNRs is how they report on the global indicators. High-income countries have particular issues in identifying and reporting on health inequities, as their achievement of health indicators for the majority of their populations can mask deep and continuing health inequities.^55^ For instance, as is also shown in our analysis of the NVNR, numbers may easily lend themselves to so-called window-dressing; the presentation of a favourable situation for the majority that conceals sub-level inequalities.^56,57^ In the NVNR, indicator measurements are presented indirectly and with few actual numbers, and in global comparison. This strategy is also noted in the previously mentioned debate article signed by a broad range of civil society organisations, which advocate the need for national indicators.

 Whereas well-crafted indicators can sometimes be essential to document progress or real change in policy and action,^42^our results illustrate that indicator reporting is also very much a question of how the numbers are presented. For instance, in the SVNR, global comparisons are more systematically nuanced by explications of the numbers’ limitations and specifying on national inequalities. This was also observed in the recently published strategy document “Next steps towards more equal health – Propositions for long-term work for good and equal health” (our translation),^58^ it is stated that: “Sweden is a country where public health, measured traditionally – like infant mortality or life expectancy – is very good, but where inequality in health and life expectancy is very prominent.”^58^That is, the clear limitations in global indicators can be actively counteracted to ensure discourses that maintain the Agenda’s cross-cutting focus on inequality.

 Whereas the limitations of quantifying holistic and integrated goals have been amply discussed, research on SDG implementation has so far not included a discussion of discourse development. We suggest that future research on follow up of the SDGs should pay more attention to possible implications of what might be coined a *metrics-driven discourse*, ie, a discourse that privileges, and is organized around quantitative measurements, instructed and restricted by the categories and results of quantitative and statistical operations. An example would be the description of health in the NVNR, where the indicators (rather than the SDG goals) seem to determine priorities, displacing the carefully negotiated language^49^ of the 2030 Agenda, and reducing the holistic conceptualization of health into the sum of isolated fragments. We argue that such discourses may potentially fuel fragmentation instead of integration, and provide weak grounds for integrated policy development. Accordingly, as demonstrated by our analysis of the SVNR, framing health in an inequality oriented perspective seems to underscore the need for governmental leadership in novel cross-sectoral initiatives. As our results demonstrate, with added interpretation and contextual comment a fuller, richer picture can emerge – the VNRs can serve as important vehicles for the establishment of discourses that avoid known mechanisms of exclusion, simplification and selectivity in reporting. Discourses and norms can effectively drive or restrict change. Further research should address the development of implementation discourses in a wider set of VNRs.

 While our results from the two selected VNRs are not a sufficient sample to draw broad conclusions, they do illustrate how different framings of health reflect differing understandings of health and point to varying potential actions to achieve the SDGs. As such, our results provide a basis for further research to investigate the implications of these frames for policy and action. For instance, our results prompt the question of which measures Norwegian health policy in the wake of the 2030 Agenda will take to ensure that no one “is left behind.” Another interesting question to pursue is if and how competing framings of health in the two VNRs will influence development of health policy to achieve the SDGs in these countries. A further question to ask is whether the different frames used in the two documents reflect differing commitments to implementing the SDGs domestically, or other political concerns, such as seeking to project a particular image internationally, as described by Bexell and Jönsson.^42^ The UN 2030 Agenda is an ambitious inter-sectoral agenda for all countries, including high-income countries, to work toward a more sustainable and equitable future for their populations. This paper has used frame analysis to investigate and compare how health is framed in the VNRs of two high-income countries, Norway and Sweden, and the implications the frames used have for potential actions by these nations. The main findings are that the NVNR has framed health with a focus on the favourable situation of the majority, with little attention to inequities; while the SVNR makes health inequities and social issues central to its framing and discussion of health. This comparison highlights the importance of analysing discourses and frames used in VNRs as a guide to the extent (or lack) of implementation nations may embark upon, offering additional contextual understandings not generally offered by a metrics-driven evaluation framework. To realise the 2030 Agenda’s vision of ‘leaving no one behind,’ discourses of implementation that support the Agenda’s inclusive and holistic ambition are needed. Policy-makers can utilize the presented results to ensure that future framings of health adequately reflects the 2030 Agenda’s transformative ambition and focus on leaving no one behind. Further development of the follow-up and review system should acknowledge and address how frames can limit or enable integrative actions and are therefore important drivers of change.

## Acknowledgements

 This article was written as part of the international research program on “The Body in Translation: Historicising and Reinventing Medical Humanities and Knowledge Translation” at the Centre for Advanced Study at the Norwegian Academy of Science and Letters in Oslo during the academic year 2019/2020.

## Ethical issues

 The study’s material consist of publicly available government documents and is exempted from evaluation by ethics committees.

## Competing interests

 Authors declare that they have no competing interests.

## Authors’ contributions

 IL designed the study, developed analysis and drafted the manuscript. KMH contributed substantially to the study design, analyses, and critical revision of the manuscript. GT contributed substantially to the analysis and critical revision of the manuscript. EE contributed substantially to the study design, analyses, and critical revision of the manuscript.

## Funding

 This work was funded by Centre for Sustainable Health Care Education, University of Oslo, Norway.

## Authors’ affiliations

^1 ^Centre for Sustainable Health Care Education, University of Oslo, Oslo, Norway. ^2^Swedish Institute for Global Health Transformation (SIGHT), Royal Swedish Academy of Sciences and Presidents Office, Stockholm, Sweden. ^3^Karolinska Institutet Stockholm, Stockholm, Sweden.

## Supplementary files

Supplementary file 1. SDG 3 List of Targets.
