# Supplementary material for: Implementing the UN Sustainable Development Goals: How Is Health Framed in the Norwegian and Swedish Voluntary National Review Reports?
Source: Int J Health Policy Manag. 2020 Nov 23;11(6):810–9. doi: 10.34172/ijhpm.2020.221 (PMC9309910; doi:10.34172/ijhpm.2020.221)
Supplement: Supplementary file 1 — SDG 3 List of Targets. [file ijhpm-11-810-s001.pdf]

Supplementary file 1. SDG 3 List of targets

SDG 3 Ensure healthy lives and well-being for all at all ages
3.1 By 2030, reduce the global maternal mortality ratio to less than 70 per 100 000 live births.<br><br>3.2 By 2030, end preventable deaths of newborns and children under 5 years of age, with all countries aiming to reduce neonatal mortality to at least as low as 12 per 1,000 live births and under-5 mortality to at least as low as 25 per 1000 live births.<br><br>3.3 By 2030, end the epidemics of AIDS, tuberculosis, malaria and neglected tropical diseases and combat hepatitis, water-borne diseases and other communicable diseases.<br><br>3.4 By 2030, reduce by one third premature mortality from non-communicable diseases through prevention and treatment and promote mental health and well-being.<br><br>3.5 Strengthen the prevention and treatment of substance abuse, including narcotic drug abuse and harmful use of alcohol.<br><br>3.6 By 2020, halve the number of global deaths and injuries from road traffic accidents.<br><br>3.7 By 2030, ensure universal access to sexual and reproductive health-care services, including for family planning, information and education, and the integration of reproductive health into national strategies and programmes.<br><br>3.8 Achieve universal health coverage, including financial risk protection, access to quality essential health-care services and access to safe, effective, quality and affordable essential medicines and vaccines for all.<br><br>3.9 By 2030, substantially reduce the number of deaths and illnesses from hazardous chemicals and air, water and soil pollution and contamination.<br><br>3.A Strengthen the implementation of the WHO Framework Convention on Tobacco Control in all countries, as appropriate.<br><br>3.B Support the research and development of vaccines and medicines for the communicable and noncommunicable diseases that primarily affect developing countries, provide access to affordable essential medicines and vaccines, in accordance with the Doha Declaration on the TRIPS Agreement and Public Health, which affirms the right of developing countries to use to the full the provisions in the Agreement on Trade Related Aspects of Intellectual Property Rights regarding flexibilities to protect public health, and, in particular, provide access to medicines for all.<br><br>3.C Substantially increase health financing and the recruitment, development, training and retention of the health workforce in developing countries, especially in least developed countries and small island developing States.<br><br>3.D Strengthen the capacity of all countries, in particular developing countries, for early warning, risk reduction and management of national and global health risks.
